# Blood in Capsules: Multi-Technique Forensic Investigation of Suspicious Food Supplement

**DOI:** 10.3390/molecules30234600

**Published:** 2025-11-29

**Authors:** Paweł Rudnicki-Velasquez, Magdalena Popławska, Karolina Pioruńska, Marta Łaszcz, Małgorzata Milczarek, Anna Pogorzelska, Michał Karyński, Agata Błażewicz

**Affiliations:** 1 Falsified Medicines and Medical Devices Department, National Medicines Institute, Chełmska 30/34, 00-725 Warsaw, Poland; m.poplawska@nil.gov.pl (M.P.); k.piorunska@nil.gov.pl (K.P.); m.laszcz@nil.gov.pl (M.Ł.); m.karynski@nil.gov.pl (M.K.); a.blazewicz@nil.gov.pl (A.B.); 2 Biomedical Research Department, National Medicines Institute, Chełmska 30/34, 00-725 Warsaw, Poland; m.milczarek@nil.gov.pl (M.M.); a.pogorzelska@nil.gov.pl (A.P.)

**Keywords:** multi-technique workflow, forensic analysis, illicit food supplements, heme detection, biological adulteration, scoping review

## Abstract

This study presents the results of a multi-technique forensic investigation of suspicious soft capsules seized by law enforcement during a criminal case. The unlabeled samples, sold as therapeutic and “regenerative” food supplements, were examined using liquid chromatography–tandem mass spectrometry (LC-MS/MS), Fourier-transform infrared spectroscopy with attenuated total reflection (ATR-FTIR), chemiluminescence, and brightfield/confocal microscopy. These complementary analytical approaches revealed that the capsules contained biological material of unknown origin, including blood-derived compounds, lipid constituents, and cellular structures. The findings indicate biological adulteration, possibly due to deliberate falsification or severe contamination. To place these results in a broader biomedical context, a scoping review of literature on blood- and tissue-derived materials used in biomedical and nutraceutical applications was conducted. This review underscores how such products are developed, promoted, and regulated, highlighting the potential health and biosafety risks associated with unregulated biologically themed supplements. Overall, this study demonstrates a transferable analytical workflow suitable for forensic laboratories and emphasizes the need for continued regulatory vigilance to protect public health. Given the evidentiary constraints typical of forensic casework—specifically, the small amount of seized material—the workflow was optimized to maximize information yield through minimally destructive, orthogonal, non-genetic screening methods, with LC-MS/MS reserved for final molecular confirmation. DNA typing was not performed because, after confirmatory analyses, the remaining material was insufficient for reliable genotyping.

## 1. Introduction

Globally, the falsification of pharmaceuticals and food supplements has become a persistent public health concern [1]. Counterfeit or adulterated products may contain undeclared active ingredients, insufficient dosages, or harmful contaminants. Each of these scenarios can lead to treatment failure or unexpected adverse effects, increasing health risks [2,3,4]. Regulatory systems are in place, but they are struggling to keep up. The rapid growth of online sales and cross-border trade has outpaced conventional control measures, especially for supplements promoted as “natural” or “traditional” [5]. The diversity of such products, combined with poor labeling and inconsistent quality control, makes detection and enforcement even more challenging. In addition, the growing popularity of biologically derived ingredients introduces new analytical and biosafety challenges, which require specialized approaches to ensure authenticity and consumer safety [6].

In recent years, the nutraceutical and cosmetic industries have increasingly turned to unusual biological sources, including placental extracts, blood derivatives, and deer antler velvet [7,8]. These products are typically marketed as rich in bioactive molecules—proteins, peptides, growth factors-like components, or steroidal hormones—claimed to enhance vitality, regeneration, or sexual performance [8,9]. Their commercial appeal is clear, but clinical evidence to support such claims is often weak. Their use also raises ethical and regulatory concerns, including the potential transmission of pathogens or inadvertent exposure to hormonally active compounds [9,10,11].

Regulatory agencies in different parts of the world have drawn attention to the marketing of supplements that promise effects far beyond what can be supported by evidence. In the United States, the Food and Drug Administration (FDA) has repeatedly issued consumer alerts on so-called “regenerative medicine products,” including stem cell and exosome preparations, stressing that they have never been proven effective and in some cases may expose patients to serious harm [12]. State authorities have reinforced these warnings: the Illinois Department of Public Health, together with the Centers for Disease Control and Prevention (CDC), advised patients after bacterial infections were reported in users of contaminated stem cell products, illustrating how easily unregulated preparations can become vehicles for pathogens [13]. Comparable problems have been documented in Asia. The Health Sciences Authority (HSA) in Singapore successfully prosecuted the distributor of Purtier Placenta for falsely presenting the product as a stem cell therapy for cancer and diabetes [14]. International media have also contributed to public awareness: AFP journalists debunked viral social-media adverts that described “live stem cell pills” as universal cures, pointing out that such capsules contain no viable cells and cannot deliver the promised effects [15]. These examples illustrate that the phenomenon is global and underline why laboratory methods capable of authenticating suspicious products are urgently needed.

In Poland, law enforcement authorities have reported a rising number of suspicious supplements submitted for forensic analysis. Capsules and tablets advertised as “natural remedies”, or “regenerative supplements” are increasingly encountered on the black market. Many deviate from their declared composition, and some contain substances of unknown or illicit origin. The case analyzed here is not an isolated exception, but part of a broader trend observed in routine forensic casework [16]. These developments underline the need for analytical workflows that can rapidly and credibly identify adulteration, providing evidence suitable for both regulatory decisions and judicial proceedings.

In this study, we describe such a workflow. We combined several complementary techniques—liquid chromatography–tandem mass spectrometry (LC-MS/MS), attenuated total reflection Fourier-transform infrared spectroscopy (ATR-FTIR), chemiluminescence, and digital/confocal microscopy—to examine capsules seized during a forensic investigation. This analytical combination was selected to capture different levels of structural information: chemiluminescence served as a rapid presumptive test for heme-related peroxidase activity, ATR-FTIR provided molecular fingerprinting of organic functional groups, LC-MS/MS enabled high-specificity confirmation of low-molecular-weight biomarkers such as heme B, and microscopy supplied morphological evidence of cellular remnants. Together, these complementary techniques formed an orthogonal workflow capable of verifying biological adulteration even in highly complex or degraded matrices. The different methods converged on the same conclusion, revealing heme-related compounds and cellular remnants that point to the presence of undeclared biological material.

## 2. Ethical Statement

The material analyzed in this study was provided by the Polish Public Prosecutor’s Office as part of an official forensic investigation. All samples were anonymized and not linked to identifiable individuals, meaning that no personal or clinical data were involved. Our analyses were limited to the chemical and microscopic characterization of the capsule contents, and no experiments were conducted on humans or animals. According to institutional and national guidelines for forensic casework, separate approval from an ethics or bioethics committee was therefore not required.

## 3. Results and Discussion

**Figure 1:**
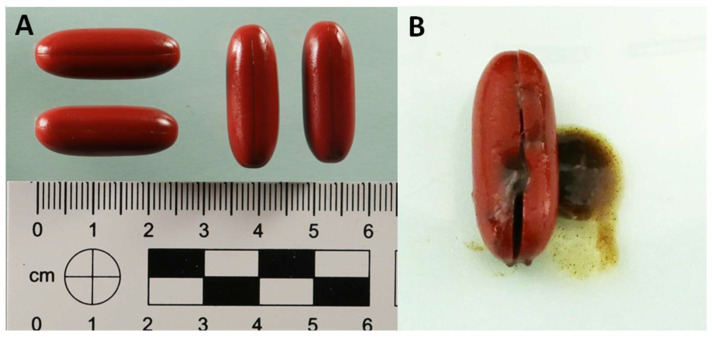
External appearance of capsules (**A**) and internal content of a capsule after incision (**B**).

Figure 1A illustrates the external appearance of the capsules. They were softgel, opaque and uniformly shaped, measuring approximately 2.5 cm in length, with a dark red color. As is typical of gelatin-based formulations, the surface was smooth and elastic. Notably, there were no imprints, logos, batch codes or regulatory markings, which made it impossible to trace their origin or identify the manufacturer. When a capsule was cut open (Figure 1B), the inside looked very different. The contents formed a dense, viscous suspension that was dark red-brown in color and had a heterogeneous texture. Within this matrix, small aggregates and sediment-like particles were clearly visible, suggesting the presence of complex biological or lipid-derived material. The striking contrast between the uniform exterior and irregular interior indicated a non-homogeneous formulation. Such macroscopic features are unusual for conventional softgel products, which typically contain a clear suspension or oil. In standard pharmaceutical and nutraceutical softgels, the fill material is homogeneous, optically transparent or uniformly opaque, and remains fluid or semi-fluid to ensure even distribution of active ingredients within a gelatin shell [17,18]. The presence of a solidified, granular, and phase-separated core-as observed here-is inconsistent with the manufacturing and physical characteristics of legitimate softgel formulations. This discrepancy strongly suggested adulteration of the capsule fill material, particularly given how atypical the morphology is for legitimate softgel formulations.

**Figure 2:**
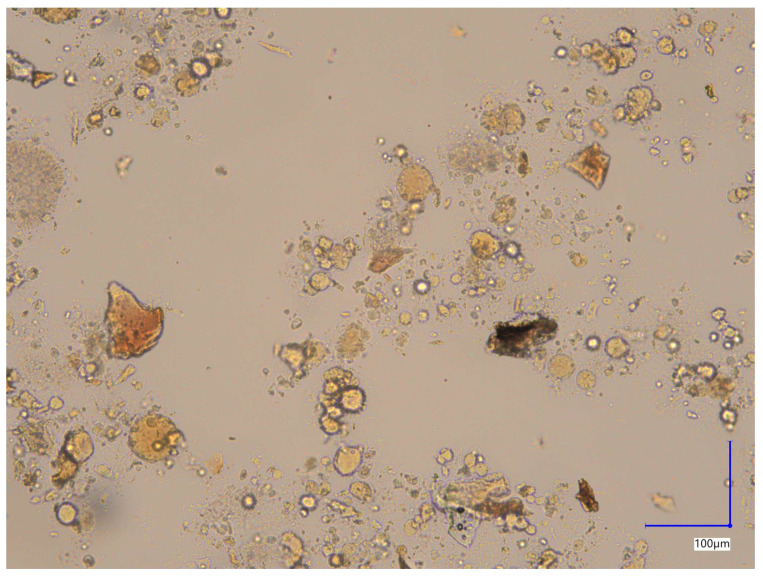
Brightfield image of unstained capsule content.

Brightfield image of the unstained capsule contents (Figure 2) reveals heterogeneous morphology of particles with respect to their shape and size. Observed features included single rounded, elongated and irregular forms or their aggregates.

**Figure 3:**
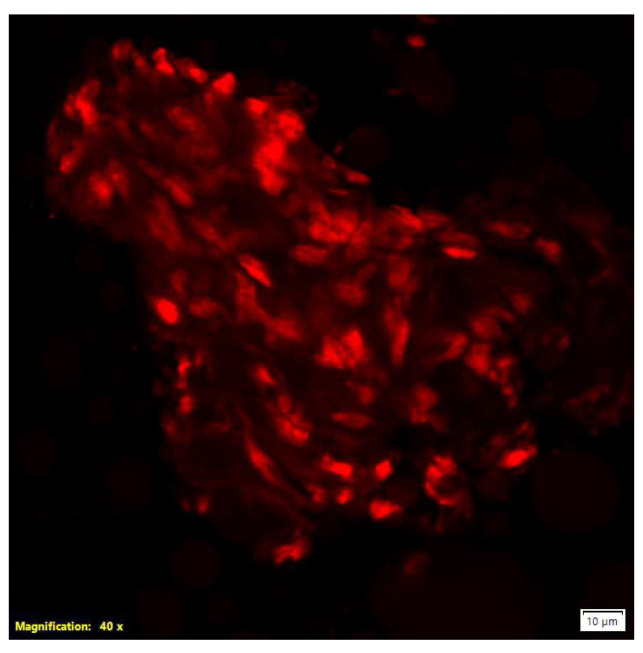
Three-dimensional visualization of the nucleus in cell aggregates using a confocal microscope. Red fluorescence indicates the nucleus; scale bar: 10 µm; magnification: 40×.

Confocal microscopy answered the question that some of particles have biological origin. This microscopy using propidium iodide (PI) staining demonstrated red fluorescent nuclei indicative of cells with compromised plasma membranes. PI selectively intercalates into the nucleic acids of cells exhibiting loss of membrane integrity, making it a well-established marker of cell death. Additionally, microscopic observations revealed the formation of aggregates by cells or cellular components (Figure 3) [19].

We observed an immediate blue light emission when the luminol–H_2_O_2_ reagent was applied to the capsule contents spread on fabric. This classic chemiluminescent response points to the presence of compounds with peroxidase-like activity. Such activity is usually linked to hemoproteins, such as hemoglobin, although it is not entirely specific. When viewed alongside the microscopy findings—which showed structures resembling eukaryotic cell remnants—the luminol reaction provided preliminary evidence that blood-derived material was present [20]. At the same time, we emphasize the limitations of this test. Because luminol is inherently non-specific, the assay should be considered only presumptive. Definitive identification of the catalytically active compounds requires confirmation with more selective analytical techniques.

The ATR-FTIR spectrum of the capsule contents showed clear absorption bands that are typical of glycerol esters of fatty acids [21]. In simple terms, this indicates the presence of various oils, although the data did not enable us to identify a specific type (Table 1). A library search corroborated this finding, revealing similarities to several common edible oils, including nut oils, grape seed oil, and olive oil. After centrifugation and removal of the oily fraction, we examined the spectrum of the remaining dry residue. Some changes were visible, but they were insufficient to enable the solid components to be identified with certainty. Overall, the mixed signals observed suggest two possibilities: either oils were deliberately added as excipients, or the material was contaminated with lipid-rich biological matter.

To enhance the analytical proof, the corresponding ATR-FTIR spectrum of the capsule content is provided in the Supplementary Materials (Figure S1), together with library-based overlays and reference matches for representative edible oils (Figure S2). These comparative overlays confirm the predominance of triglyceride-type bands, consistent with lipid excipients as listed in Table 1. No distinct Amide I–II bands (1650–1550 cm^−1^) were observed, and all top library matches corresponded to edible oils and fats, indicating a predominantly lipid matrix. This observation aligns with LC-MS/MS evidence of minor heme-related residues rather than bulk protein.

**Figure 4:**
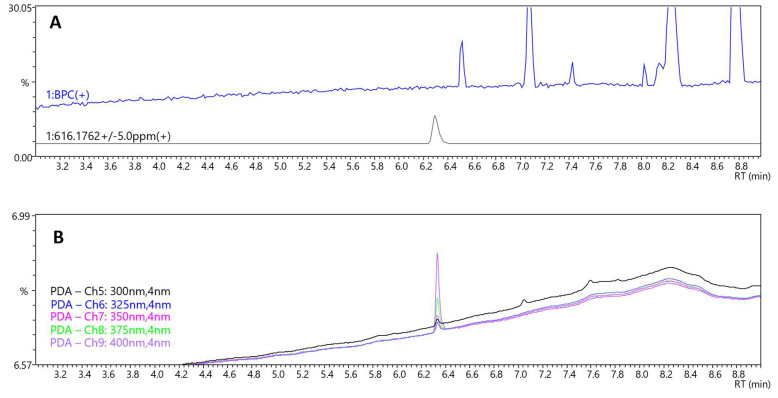
Chromatograms recorded for the sample; MS chromatograms: base peak chromatogram (BPC) with extracted-ion chromatogram (EIC) (616.1762) (**A**); PDA chromatogram with extracted wavelengths 300 nm, 325 nm, 350 nm, 375 nm, 400 nm (**B**).

**Figure 5:**
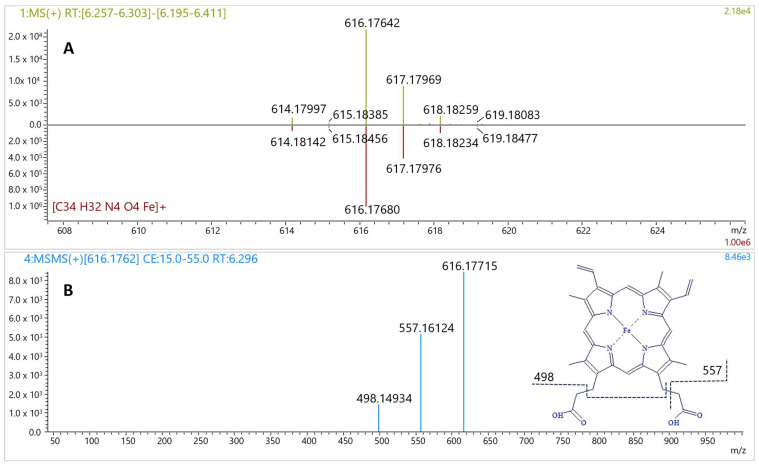
MS spectrum of the peak at RT = 6.3 min compared with theoretical MS spectrum (**A**); product ion spectrum (MS/MS) of the precursor ion at *m*/*z* 616.1762 with proposed fragmentation scheme (**B**).

For LC-MS/MS analysis, a comprehensive screening protocol was implemented to detect and characterize constituents within the capsule matrix, the composition of which was otherwise undocumented. Although no specific peaks (not originating from solvent) except lysophosphatidylcholine, glyceryl caprylate dicaprate, and glyceryl caprate dicaprylate were found on the LC-MS/MS base peak chromatogram (BPC) (Figure 4A and Figure S4, Supplementary Materials), a distinct peak at RT = 6.3 min was observed on the LC-PDA chromatogram. The intensity of this peak increased with wavelength from 300 to 400 nm (Figure 4B). The corresponding MS spectrum showed a signal at *m*/*z* 616.17642 (Figure 5A), consistent with the molecular formula C_34_H_32_FeN_4_O_4_ (calculated: 616.17674, error −0.52 ppm, IsoScore 90%). The isotopic pattern was highly distinctive due to the presence of an iron atom in the molecule (Figure 5A). The MS/MS spectrum of this precursor ion yielded fragments at *m*/*z* 498.14934 and 557.16124 (Figure 5B), characteristic for the fragmentation of heme B [22]. These product ions are consistent with classical heme B MS/MS behavior reported in high-resolution studies, which commonly show fragments at *m*/*z* 557 and 498 for heme under positive-mode CID. In *Rhodnius prolixus*, Paiva-Silva et al. observed the same ions upon fragmentation of the *m*/*z* 616 heme ion and of heme-derived intermediates, supporting their diagnostic value for the Fe-porphyrin core [23]. The matching fragmentation pattern in our sample substantiates assignment of the *m*/*z* 616.176 precursor to heme B rather than to a related tetrapyrrole. Together with the maximum absorbance observed at 400 nm, very close to the reported absorption maximum of heme B [24], these data confirm the presence of heme B in the capsule content. Although heme B can also occur in plants, its biochemical context is fundamentally different. In plant tissues, heme is almost entirely protein-bound within plastids, and only a very small fraction exists in a free form that can be extracted without protein denaturation [25]. In this study, heme B was detected directly in the extract without proteolytic treatment, and the LC-MS/MS data (precursor *m*/*z* 616.176, isotopic pattern, and diagnostic fragments) were consistent with free heme rather than protein-bound complexes. Such a spectral and structural profile is typical of blood-derived residues, whereas plant matrices generally yield only trace heme signals under non-denaturing conditions. Collectively, these observations are most consistent with a blood-derived rather than plant origin of the detected material. The molecular data definitively confirmed the chemiluminescence results and corroborated the microscopic observations of cellular remnants. Taken together, these results provide convergent evidence that the material is of blood-derived origin. The detection of heme B, together with the microscopic and chemiluminescent findings, is inconsistent with a purely plant- or excipient-based composition and most plausibly indicates the presence of blood-derived residues. Understanding the biological nature of the material is not just a technical detail—it is essential for appreciating the toxicological and biosafety risks associated with consuming such a product.

In this case, the main toxicological concern is the potential infectivity of the contents of the capsule. The presence of cellular material of unknown origin raises concerns about pathogen transmission. This includes not only common blood-borne pathogens, but also—at least in theoretical terms—agents associated with transmissible spongiform encephalopathies. There is a clear difference here with conventional animal-derived food products, which must undergo veterinary inspection and comply with food safety regulations. By comparison, biological preparations sold as therapeutic or ‘regenerative’ supplements should adhere to even stricter standards of sterility, traceability, and pathogen screening. In this case, however, none of these guarantees were in place. This absence underscores just how serious the risks are when consuming unregulated biological formulations. Comparable observations were previously reported in analyses of placenta- or stem-cell-based supplements, where microscopy and biochemical assays similarly revealed undeclared biological residues rather than the claimed active cells [26]. This indicates that our case is part of a broader phenomenon of fraudulent biologically themed products.

Beyond the direct biomedical risks, our findings raise important social and ethical concerns. Falsified food supplements tend to target people who are already vulnerable-for example, patients with terminal or life-threatening illnesses—who may be especially drawn to promises of regenerative therapies. For such people, even the suggestion of renewed health can hold a strong psychological appeal. The result is often heavier financial and emotional burdens, combined with a loss of trust in legitimate medicine. These realities point to a dual imperative. On the one hand, we need analytical workflows that can reliably detect biologically adulterated products. On the other hand, stronger regulatory, educational, and ethical safeguards must be in place to limit their spread. Our study contributes a practical pathway for forensic laboratories, while also underlining the urgent societal need for vigilance against the return of unregulated, biologically hazardous therapeutics.

These concerns are echoed in international regulatory and public health reports. The FDA has emphasized that stem cell and exosome products marketed as “regenerative” remain unapproved and unproven, with risks ranging from false hope to serious adverse reactions [12]. The Illinois Department of Public Health and the CDC issued warnings after infections were linked to stem cell preparations produced without proper controls [13]. In Singapore, the HSA took legal action against misleading advertising of Purtier Placenta, which had been promoted as an anti-cancer stem cell therapy despite the absence of any scientific basis [14]. Independent investigations have confirmed the same pattern in public communication: Agence France-Presse (AFP), for example, demonstrated how “live stem cell pills” were promoted across Southeast Asia with sweeping promises of cures for chronic diseases, even though scientific analysis showed that the capsules contain no live cells [15]. Taken together, these reports make clear that the capsules examined here are not an isolated curiosity but part of a broader and persistent problem. Our case therefore not only demonstrates the practical value of a multi-technique forensic workflow but also places it within the global context of fraudulent, biologically themed supplements.

Our observations also corroborated previous reports on biologically themed supplements. In their study, Santos et al. [26] examined capsules that were declared to contain live deer placenta stem cells. Using orthogonal methods such as cytology, histology, immunohistochemistry, flow cytometry and electron microscopy, they found no viable stem cells. Instead, a mixture of lipids, proteins, collagen, bacteria and crystalline material was detected. Taken together, our findings and those previously described revealed the twofold nature of this fraudulent practice. Some products claim to contain biologically active cells but do not, while others contain undeclared blood-derived residues that pose potential biosafety risks. Both cases highlight the importance of multi-technique forensic workflows in assessing such preparations, exposing false or dangerous claims, and protecting patients.

## 4. Scoping Review of Blood and Tissue-Derived Products in Biomedical and Nutraceutical Applications

To better place our forensic findings in context, we looked at the available literature on the use of blood- and tissue-derived materials in both biomedical research and commercial food supplements. We searched PubMed and Scopus for publications from 2010 to 2024 using keywords such as platelet lysate, human serum supplement, placenta extract, deer antler, and stem cell supplement. We included not only primary studies-such as in vitro experiments, animal studies, and randomized clinical trials-but also systematic and narrative reviews, when they represented the best available evidence in a given area. For example, in the case of velvet antler supplements, comprehensive systematic reviews were cited rather than repeating details of each small clinical trial. Eligible studies were those that investigated human- or animal-derived materials in biomedical or nutraceutical settings and that reported on composition, functional effects, or safety. Only English-language publications were considered. From each study we noted the type of material, its intended use, the study design, the main results, and any discussion of safety or regulation. The findings are presented narratively and summarized in a table for clarity.

Several groups have examined platelet lysate (PL) and human serum (HS) as potential alternatives to fetal bovine serum for cell culture. Phetfong et al. [27] and Suchankova Kleplova et al. [28] reported that human PL supports proliferation and preserves multipotency of mesenchymal and dental pulp stem cells. Arpornmaeklong et al. [29] observed similar effects with allogeneic human serum, and Naskou et al. [30] reported that pooled equine platelet lysate can replace fetal bovine serum (FBS) as a serum-free supplement for in vitro expansion of equine bone-marrow mesenchymal stem cells (MSCs) while preserving phenotype, trilineage differentiation, and immunomodulatory activity. Collectively, these studies indicate that, when prepared and screened under standardized conditions, blood-derived supplements can serve as viable alternatives for specific laboratory applications. Extending this line of work, Kim et al. [31] tested a human embryonic stem cell-derived bioactive material in bovine embryo culture and observed improved blastocyst development. These examples illustrate that biological derivatives are being developed within biomedical science under controlled conditions with attention to biosafety.

The picture is quite different when it comes to nutraceuticals marketed directly to consumers. Velvet antler from deer or elk is widely sold as a food supplement meant to boost vitality or treat chronic conditions. Yet systematic reviews and clinical trials provide little support for these claims. Gilbey and Perezgonzalez [8] reviewed the available randomized trials and found no convincing benefits in arthritis, sexual function, or athletic performance. A large placebo-controlled trial by Allen et al. [32] also failed to show significant benefit in rheumatoid arthritis patients. Some preclinical studies do suggest potential biological activity: Yao et al. [33] reported effects on cartilage gene expression in rats, Chen et al. [34] found modest bone-related changes in rodents, Yu et al. [35] identified enzyme-inhibiting peptides from antler proteins, and Ren et al. [36] described osteoprotective effects in an animal osteoporosis model. Still, these remain laboratory or animal findings, and so far, they have not translated into reliable evidence of human benefit.

Placental preparations fall between regulated biomedical research and commercial supplements. Reviews by Pan et al. [7] and by Pogozhykh et al. [37] summarize the rich content of placental extracts (growth factors, nucleic acids, extracellular proteins) and exploratory use in wound healing and regenerative models; but robust clinical validation is still lacking. Encapsulated human placenta—sometimes promoted as “placentophagy”—has also entered the supplement market, but reviews report no consistent evidence for postpartum recovery or mood benefits and raise safety concerns, including risks of microbial contamination, possible pathogen transmission, and heavy-metal content [38]. By contrast, regulated nutritional interventions such as omega-3 fatty acids, probiotics, or L-arginine can modulate aspects of placental biology under clinical oversight [39]; these differ fundamentally from unlabeled or black-market capsules.

Overall, the literature spans a wide spectrum. At one end are carefully standardized blood-derived products such as platelet lysates and serum, which are explored as legitimate laboratory supplements. At the other are commercial nutraceuticals like velvet antler, popular but lacking convincing clinical evidence. Placental extracts fall in between, blending traditional uses with biomedical experimentation but still raising questions about safety and standardization. Across all these categories, the key issue is the balance between biological plausibility and biosafety. Where regulation and traceability are in place, risks are minimized. Where they are absent—as with the unlabeled capsules we examined—the hazards become significant. This review underlines the importance of robust forensic tools and regulatory oversight to identify and control biologically adulterated products.

To maintain focus on the forensic casework, only a condensed synthesis is presented here; the main findings are summarized in Table 2. This scoping review is included solely to provide scientific and regulatory context for the detected biological residues and to compare legitimate biomedical applications of blood- and tissue-derived materials with their unregulated appearance in illicit preparations.

Beyond the regulated biomedical and nutraceutical uses described earlier, many peer-reviewed papers and surveillance reports have drawn attention to safety problems linked to contaminated or adulterated dietary supplements. Cohen et al. examined 21 products labeled as *Acacia rigidula* and found that more than half contained β-methylphenylethylamine (BMPEA)—a positional isomer of amphetamine with no established human safety profile. Detected levels reached up to 94 mg per daily dose, high enough to suggest pharmacological activity. Because BMPEA was not listed on the labels, the authors called for regulatory action due to its potential cardiovascular and neuropsychiatric risks [40]. Buser et al. later described a case of late-onset *Streptococcus agalactiae* infection in an infant after the mother had ingested encapsulated, dehydrated placenta. Identical bacterial strains were isolated from both the infant’s blood and the capsules, demonstrating vertical transmission; the dehydration process (46–71 °C) had failed to eliminate pathogens, prompting the CDC to advise against placenta capsule consumption [41]. Other biologically derived supplements have shown similar toxicological issues. Saper et al. found that roughly one-fifth of 193 Ayurvedic preparations sold online contained lead, mercury, or arsenic above international limits, with contamination present in both U.S.- and India-manufactured products. The highest levels were seen in *rasa shastra* formulations, which intentionally include metals, revealing persistent hazards despite “Good Manufacturing Practice” claims [42]. Tucker et al. [43] reviewed FDA warning letters issued between 2007 and 2016 and identified 776 dietary supplements adulterated with undeclared pharmaceuticals-mostly products marketed for sexual enhancement, weight loss, or muscle building. Common adulterants included sildenafil, sibutramine, and anabolic steroids, and over 20% contained more than one drug, exposing major regulatory weaknesses. Brykman et al. [44] writing in the AMA Journal of Ethics, pointed out that under the Dietary Supplement Health and Education Act (DSHEA, 1994), manufacturers must follow basic manufacturing guidelines but are not required to meet United States Pharmacopeia-National Formulary standards. As a result, products carrying identical labels may differ widely in purity and composition. The authors advised clinicians to ask patients about supplement use and to recommend only third-party-verified brands. Similarly, White’s [45] analysis of the FDA Tainted Supplement Database (2007–2021) confirmed that undeclared active pharmaceutical ingredients remain widespread. Among 1068 adulterated supplements, phosphodiesterase-5 inhibitors and sibutramine—withdrawn because of cardiovascular toxicity—were the most common, along with phenolphthalein, fluoxetine, and unapproved sibutramine analogs. Jagim et al. [46] found that between 14% and 50% of sports supplements contained anabolic or otherwise banned substances, creating a risk of accidental doping and adverse health effects; they emphasized the need for third-party certification and regular verification against prohibited-substance lists. Finally, Jairoun et al. [47] examined 277 supplements available in the UAE and detected trace levels of cadmium, lead, and arsenic. Although below the acceptable daily intake, such contamination may still pose a cumulative hazard when several products are used simultaneously. Contamination levels varied by formulation type, dosage form, and country of origin, underlining the importance of tighter surveillance and manufacturing control. Taken together, these studies clearly show that contamination and adulteration of biologically derived and dietary supplements are common and well-documented problems, highlighting the forensic, toxicological, and public-health relevance of the present case.

## 5. Materials and Methods

### 5.1. Sample

The material under investigation consisted of several soft capsules provided to the Polish Official Medicines Control Laboratory by the public prosecutor’s office as part of a forensic inquiry. The capsules had been seized during an investigation into the distribution of unregulated food supplements promoted as cures “for all diseases”. No manufacturer details, leaflets, batch numbers, or quality certificates were available. All samples were softgel capsules, opaque, and uniformly shaped, containing a viscous dark red–brown suspension that immediately raised concerns of possible biological adulteration. After receipt, the capsules were stored at 4 °C until analysis. The number of capsules provided by the prosecutor’s office was strictly limited, as they constituted evidentiary material in an active criminal investigation. No additional capsules beyond those initially seized could be obtained, and unused portions of the material could not be re-opened or consumed once the analytical workflow had been completed. These constraints prevented the performance of quantitative assays, replicate measurements, or method-validation experiments and required a workflow optimized to extract maximum evidential information from minimal sample volumes. Comparable, products submitted by law enforcement in previous cases were marketed under names such as Purtier Placenta or other so-called placenta- or stem cell-based preparations. These are typically advertised as containing placental extracts or even stem cells, yet they lack regulatory approval or verifiable documentation and are often sold at a very high price.

### 5.2. Analytical Techniques

Brightfield microscopy of the unstained suspension was performed using a Keyence VHX-X1 series digital microscope (Keyence Corporation, Osaka, Japan).

Confocal microscopy experiments were conducted on samples diluted 1:1 with phosphate-buffered saline (PBS; pH 7.4; Ca^2+^/Mg^2+^-free). Propidium iodide (PI; 4 µL per sample) was added to each tube, followed by brief vortex mixing. Samples were then incubated for 15 min at room temperature in the dark. Unstained controls were processed in parallel without dye to assess intrinsic autofluorescence and to establish acquisition parameters. After incubation, suspensions were mounted on glass microscope slides, covered with coverslips, and the coverslip edges were sealed to minimize evaporation during imaging. Imaging was performed using an Olympus Fluoview FV500/IX70 (Olympus Corporation, Tokyo, Japan) confocal inverted microscope equipped with a 40× objective. PI-stained specimens were acquired as z-stacks in confocal mode with 543 nm excitation and presented as maximum intensity projections. Acquisition settings were optimized on unstained controls and maintained constant throughout each experiment. Three technical replicates were processed for each condition.

The luminol-based reagent was freshly prepared just before the analysis began. The stock solution was prepared by dissolving 98.75 mg of luminol and 6.094 g of potassium hydroxide in deionized water and then adjusting the final volume to 100 mL in a volumetric flask. A second solution was prepared by diluting 2 mL of 30% hydrogen peroxide with 18 mL of deionized water. Equal volumes (20 mL each) of the two solutions were then mixed in a spray bottle immediately prior to use. For the assay, the contents of a single capsule were placed onto a piece of sterile cotton fabric to simulate an absorbent surface. The fabric was evenly sprayed with the luminol reagent, after which the resulting chemiluminescence was monitored under low-light conditions. Because the luminol assay is inherently presumptive and can be triggered by catalysts other than heme, we used it only as supportive screening. Definitive confirmation relied on LC-MS/MS detection of heme B.

ATR-FTIR spectra were collected using a Nicolet iS5 spectrometer (Thermo Fisher Scientific Inc., Waltham, MA, USA) fitted with a single-bounce diamond ATR accessory. Each measurement covered the 4000–400 cm^−1^ range, with 32 scans acquired per sample at a resolution of 4 cm^−1^. Background correction was performed against air. To avoid cross-contamination, the ATR crystal was cleaned with 96% ethanol after every run. Spectral processing was performed using OMNIC software (version 9.8, Thermo Fisher Scientific Inc., Waltham, MA, USA), and the resulting spectra were compared with commercial and in-house reference libraries. The capsule’s liquid content was analyzed directly. Library matching used the software’s built-in correlation match factor.

LC-MS/MS (liquid chromatography—high-resolution tandem mass spectrometry) analysis was performed on a liquid chromatograph coupled to a quadrupole time-of-flight mass spectrometer (LCMS9050-Q-TOF, Shimadzu Corporation, Kyoto, Japan). A Shim-pack Velox C18 analytical column (150 × 2.1 mm, 1.8 μm particle size, Shimadzu) was used and the column temperature was set at 40 °C. Mobile phase was composed of (A) water-acetonitrile-formic acid (90:10:0.1, *v*/*v*/*v*) and (B) methanol-acetonitrile-formic acid (90:10:0.1, *v*/*v*/*v*). A linear gradient elution at a flow rate of 0.35 mL min^−1^ was applied. A gradient started after 2 min of elution from 40 to 80%B in 5 min, then a 5 min plateau, followed by an increase to 95%B within 3 min, then kept 7 min at 95%B and return to the initial 40%B within 2 min, followed by 4 min equilibration. The photodiode array detector (PDA) was set at a wavelength range of 190–400 nm. The MS was operated in the positive ionization mode. Electrospray ionization (ESI) source parameters were as follows: drying gas flow rate, 10.0 L.min^−1^; nebulizing gas flow rate, 3.0 L.min^−1^; heating gas flow rate, 10.0 L.min^−1^; interface temperature, 300 °C; desolvation line temperature, 250 °C; heat block, 400 °C; and interface voltage, 4000 V. Data Dependent Analysis algorithm with six MS/MS events and the collision energy (CE) fixed at 35 V with a CE spread of ± 20 V was selected. Data acquisition was performed over a scan range from 50 to 1500 *m*/*z* for both the MS and MS/MS events, and a sodium formate solution was used for MS calibration. Product-ion spectra were interpreted by comparison with published ESI (+)-CID fragmentation data for heme B (Fe-protoporphyrin IX). Importantly, the LC-MS/MS workflow was not a proteomics protocol; no protein extraction, enzymatic digestion, or peptide-level analysis was performed. The method was optimized specifically for the detection of low-molecular-weight compounds, including free heme. Confirmation criteria included the exact mass of the precursor ion (*m*/*z* 616.176 ± 2 ppm), the isotopic pattern consistency (IsoScore > 50%), the presence of the Soret absorbance band near 400 nm on the PDA trace, and at least two diagnostic fragments at *m*/*z* 557.16 and *m*/*z* 498.15. The sample was centrifuged, and the residue was extracted with dimethyl sulfoxide which increase heme solubility and prevent heme dimers formation [24,48]. It was then diluted two-fold with a mixture of acetonitrile/acetone (1:1, *v*/*v*) and filtered by Whatman 0.2 μm pore size polytetrafluoroethylene (PTFE) filter media (GE Healthcare, Chicago, IL, USA).

## 6. Study Limitations

This investigation focused on a single forensic case, where only a few capsules were available for testing. Because the capsules constituted unique evidentiary material that could not be re-opened or consumed beyond the primary analyses, no quantitative assays, replicate measurements, or validation experiments could be performed. The limited number of samples provided by the Prosecutor’s Office had to be divided among several analytical techniques (ATR-FTIR, chemiluminescence, microscopy, and LC-MS/MS). In such forensic work, the primary goal is qualitative confirmation of biological adulteration rather than quantification, especially when the evidence cannot be replaced. For this reason, bulk protein assays and semi-quantitative LC-MS workflows were not feasible under these conditions. DNA typing or species-specific polymerase chain reaction (PCR) was also not attempted, as the remaining material was insufficient after completing the required confirmatory tests.

The biological origin of the material was therefore inferred from several non-genetic indicators: PI-positive nuclei observed under the microscope, a luminol reaction typical of heme-catalyzed peroxidase activity, ATR-FTIR spectra indicating a lipid-based matrix, and LC-MS/MS confirmation of heme B supported by its PDA absorbance at 400 nm. A certified heme B standard was not analyzed in parallel because all evidentiary material had been consumed. Nevertheless, the compound’s accurate mass, isotopic pattern, and characteristic MS/MS fragments matched published reference data, providing high confidence in its identification.

Overall, this study demonstrates the usefulness of a multi-technique forensic approach for detecting biological adulteration, while emphasizing that the findings should be interpreted cautiously within the limits of a single-case investigation.

## 7. Green Chemistry Assessment

Alongside the analytical performance, the workflow was also considered from the viewpoint of Green Analytical Chemistry (GAC). The individual techniques differ strongly in their environmental impact. ATR-FTIR spectroscopy and optical microscopy are almost waste-free, as they require no organic solvents and produce negligible residues. The luminol chemiluminescence assay also uses only trace amounts of reagent and generates minimal alkaline waste.

In contrast, LC-MS/MS is the least environmentally friendly step, consuming organic solvents and relying on energy-intensive equipment. Even so, the tiered design of the workflow helps to balance these factors. Low-impact techniques—ATR-FTIR, microscopy, and luminol testing—are used first for rapid screening, and LC-MS/MS is applied only for final confirmation. This approach reduces the number of solvent-intensive runs and minimizes the overall environmental footprint.

The same tiered order also reflects practical casework constraints. In forensic laboratories, evidentiary samples are often very limited, so using quick and non-destructive tests first helps to preserve material for confirmatory analysis. In that sense, the workflow achieves a realistic balance between analytical certainty, sample conservation, and environmental responsibility.

## 8. Conclusions

This investigation showed that the unlabeled capsules seized from the illicit market contained biological material, including clear evidence of blood-derived compounds. By combining LC-MS/MS, ATR-FTIR, chemiluminescence, and brightfield/confocal microscopy, we established a practical, reproducible workflow for confirming biological adulteration in suspicious products. The results underline the need for stronger regulatory oversight and closer cooperation between analytical laboratories, health authorities, and law enforcement agencies to protect consumers from unverified and potentially hazardous materials. The workflow presented here can be directly applied in routine forensic or regulatory practice, providing a rapid and reliable means of identifying biological adulteration in questionable “regenerative” or therapeutic preparations.

## Figures and Tables

**Table 1:** Characteristic ATR-FTIR absorption bands of the capsule content with corresponding assignments.

Typical Wavenumber (cm^−1^)	Bands Assignment	Spectrum of Capsules Content (cm^−1^)
~3010	=C–H stretching (unsaturated fatty acids)	3009
~2950	–CH_3_ stretching	2953
~2920	–CH_2_ asymmetric stretching	2922
~2850	–CH_2_ symmetric stretching	2853
~1740–1750	C=O stretching (carbonyl group from triglycerides)	1743
~1650	C=C stretching	1653
~1465	–CH_2_ bending (scissoring)	1464
~1377	–CH_3_ symmetric bending	1377
~1160–1170	C–O stretching (ester, asymmetric)	1160
~1110–1120	C–O stretching (ester, symmetric)	1119
~720	–(CH_2_)ₙ rocking (long aliphatic chains)	722

**Table 2:** Summary of studies on blood- and tissue-derived materials relevant to biomedical and supplement contexts.

Material/Product	Application/Context	Evidence Level	Key Findings	Safety/Regulatory Concerns
Human platelet lysate (hPL)	MSC and dental pulp stem cell expansion [27,28]	In vitro, controlled	Promotes proliferation and multipotency	Requires screening, standardized preparation
Human serum (HS)	Periodontal ligament stem cell culture [29]	In vitro	Supports MSC phenotype and osteogenesis	Decline at high passages; biosafety critical
Equine platelet lysate	Veterinary MSC expansion [30]	In vitro, veterinary context	ePL supports proliferation, phenotype and trilineage differentiation; preserves TNF-α suppression vs. LPS-monocytes; potential FBS alternative	Veterinary context, not consumer
hESC-derived biomaterial	Bovine embryo culture [31]	In vitro	Enhanced blastocyst rates, pluripotency genes	Experimental, not consumer product
Velvet antler (deer/elk)	Nutraceutical (arthritis, vitality) [8,32]	RCTs, reviews	No clinical benefit beyond placebo	Widely marketed, unregulated
Deer antler extract	Rat cartilage gene modulation [33]	Preclinical, animal	ECM/collagen genes upregulated	Not validated in humans
Elk velvet antler food supplement	Rat bone development [34]	Preclinical, animal	Transient increases in body weight and bone parameters	Dosing safety unclear
Antler proteins (digested)	In vitro enzyme inhibition [35]	Preclinical, mechanistic	DPP-IV inhibition observed	Speculative clinical relevance
Pilose antler fractions	Osteoporosis model (rats) [36]	Preclinical, animal	Increased bone mineral density and osteoblast-related markers	Not validated in humans
Placenta extracts	Regenerative medicine, wound healing [7,37]	Reviews, preclinical	Growth factors identified; increased angiogenesis and tissue repair	Lack of standardization
Encapsulated human placenta	Postpartum supplement [38]	Human case reports, reviews	No proven benefits: risk of contamination reported	Microbial and heavy metal contamination
Maternal supplements (e.g., omega-3, probiotics)	Modulation of placental biology [39]	Human dietary trials	Reduced inflammation, altered transporter expression	Regulated nutritional interventions

Abbreviations: hPL—human platelet lysate; HS—human serum; MSC—mesenchymal stem cell; FBS—fetal bovine serum; hESC—human embryonic stem cell; ECM—extracellular matrix; DPP-IV—dipeptidyl peptidase-IV.

## Data Availability

All relevant data can be found within the manuscript and Supplementary Materials.

## Supplementary Materials

The following supporting information can be downloaded at: https://www.mdpi.com/article/10.3390/molecules30234600/s1, Figure S1 (ATR-FTIR spectrum of the capsule content); Figure S2 (FTIR library match for the liquid capsule content); Figure S3A–N (product-ion spectra of selected unidentified LC-MS/MS features); Figure S4 (LC-PDA chromatogram of the capsule extract).

## Abbreviations

The following abbreviations are used in this manuscript:

AFP	Agence France-Presse
ATR-FTIR	Fourier-transform infrared spectroscopy with attenuated total reflection
BPC	Base peak chromatogram
CDC	Centers for Disease Control and Prevention
DPP-IV	Dipeptidyl peptidase-IV
ECM	Extracellular matrix
EIC	Extracted-ion chromatogram
FBS	Fetal bovine serum
FDA	Food and Drug Administration
GAC	Green analytical chemistry
hESC	Human embryonic stem cell
HS	Human serum
hPL	Human platelet lysate
HSA	Health Sciences Authority
LC-MS/MS	Liquid chromatography–high-resolution tandem mass spectrometry
MSC PCR	Mesenchymal Stem Cell Polymerase Chain Reaction
PI	Propidium iodide
PL	Platelet lysate
PTFE	Polytetrafluoroethylene

## References

[1] OECD/EUIPO (2025). Mapping Global Trade in Fakes 2025: Global Trends and Enforcement Challenges. Illicit Trade.

[2] Stępień K.A., Kalicka A., Giebułtowicz J. (2024). Screening the quality of legal and illegal dietary supplements by LC-MS/MS. Food Addit. Contam. B.

[3] Pathak R., Gaur V., Sankrityayan H., Gogtay J. (2023). Tackling Counterfeit Drugs: The Challenges and Possibilities. Pharmaceut. Med..

[4] Blazewicz A., Poplawska M., Daniszewska B., Piorunska K., Karynski M. (2025). Illegal and falsified medicines self-administrated in not approved post-cycle therapy after the cessation of anabolic-androgenic steroids—Qualitative analysis. Front. Chem..

[5] Izgi G., Altınay M. (2025). Counterfeit Pharmaceuticals: Innovative Strategies for Combatting Global Health Threats.

[6] Ratajczak M., Kaminska D., Światły-Błaszkiewicz A., Matysiak J. (2020). Quality of Dietary Supplements Containing Plant-Derived Ingredients Reconsidered by Microbiological Approach. Int. J. Environ. Res. Public Health.

[7] Pan S.Y., Chan M.K.S., Wong M.B.F., Klokol D., Chernykh V. (2017). Placental therapy: An insight to their biological and therapeutic properties. J. Med. Therap..

[8] Gilbey A., Perezgonzalez J.D. (2012). Health benefits of deer and elk velvet antler supplements: A systematic review of randomised controlled studies. N. Z. Med. J..

[9] Kroløkke C., Dickinson E., Foss K.A. (2018). The placenta economy: From trashed to treasured bio-products. Eur. J. Women’s Stud..

[10] Bąk-Sypień I.I., Karmańska A., Karwowski B.T. (2019). Zafałszowania na rynku żywności funkcjonalnej i suplementów diety oraz ich potencjalny wpływ na zdrowie. Farm. Pol..

[11] Amidžić M., Banović Fuentes J., Banović J., Torović L. (2023). Notifications and Health Consequences of Unauthorized Pharmaceuticals in Food Supplements. Pharmacy.

[12] U.S. Food and Drug Administration Consumer Alert on Regenerative Medicine Products Including Stem Cells and Exosomes. FDA, 22 July 2020. https://www.fda.gov/vaccines-blood-biologics/consumers-biologics/consumer-alert-regenerative-medicine-products-including-stem-cells-and-exosomes.

[13] Illinois Department of Public Health Warning About Unproven Stem Cell Therapies–Bacterial Infections Lead to Investigation and Warning. IDPH, 8 March 2019. https://www.illinois.gov/news/release.html?releaseid=19777.

[14] Health Sciences Authority Singapore, Riway Singapore Pte Ltd. Convicted for Making False Claims that Purtier Placenta Prevents and Cures Diseases and Medical Conditions. HSA, 23 July 2021. https://www.hsa.gov.sg/announcements/news/riway-falseclaims.

[15] AFP Fact Check ‘Live Stem Cell Pills’ Promoted Online Cannot Cure Diseases: Health Experts. AFP, 16 January 2023. https://factcheck.afp.com/doc.afp.com.334X6FL.

[16] Wróbel K., Milewska A.J., Marczak M., Kozłowski R. (2022). Dietary Supplements Questioned in the Polish Notification Procedure upon the Basis of Data from the National Register of Functional Foods and the European System of the RASFF. Int. J. Environ. Res. Public Health.

[17] Gullapalli R.P. (2010). Soft gelatin capsules (softgels). J. Pharm. Sci..

[18] Podczeck F., Jones B.E. (2004). Pharmaceutical Capsules.

[19] Banfalvi G. (2017). Methods to Detect Apoptotic Cell Death. Apoptosis.

[20] Senthilkumar A., Ravindran V., Arthanari A., Ramalingam K. (2024). Evaluation of Forensic Luminol in Detection of Blood Stains in Instruments Following Dental Treatment. Cureus.

[21] Laurens L.M.L., Wolfrum E.J. (2011). Feasibility of Spectroscopic Characterization of Algal Lipids: Chemometric Correlation of NIR and FTIR Spectra with Exogenous Lipids in Algal Biomass. BioEnergy Res..

[22] Shuichi S., Mitsutoshi S. (2007). Mass Microscopy to Reveal Distinct Localization of Heme B (m/z 616) in Colon Cancer Liver Metastasis. J. Mass Spectrom. Soc. Jpn..

[23] Paiva-Silva G.O., Cruz-Oliveira C., Nakayasu E.S., Maya-Monteiro C.M., Dunkov B.C., Masuda H., Almeida I.C., Oliveira P.L. (2006). A heme-degradation pathway in a blood-sucking insect. Proc. Natl. Acad. Sci. USA.

[24] Karnaukhova E., Rutardottir S., Rajabi M., Wester Rosenlöf L., Alayash A.I., Åkerström B. (2014). Characterization of heme binding to recombinant α1-microglobulin. Front. Physiol..

[25] Espinas N.A., Kobayashi K., Takahashi S., Mochizuki N., Masuda T. (2012). Evaluation of Unbound Free Heme in Plant Cells by Differential Acetone Extraction. Plant Cell Physiol..

[26] Santos L.D., Gune S., Killingsworth M.C., Cohen-Hyams T., Wuhrer R., Harvey M., McNamara N., Nguyen L., Sabapathy S., Evangelista C. (2019). Determining and Characterizing if Deer Placenta Stem Cells Are Present in Commercial Food Supplement Capsules: Utilizing Microscopy, Elemental Analysis, Cytology, Histology, Immunohistochemistry and Flow Cytometry. Microsc. Microanal..

[27] Phetfong J., Tawonsawatruk T., Seenprachawong K., Srisarin A., Isarankura-Na-Ayudhya C., Supokawej A. (2017). Re-Using Blood Products as an Alternative Supplement in the Optimisation of Clinical-Grade Adipose-Derived Mesenchymal Stem Cell Culture. Bone Jt. Res..

[28] Suchankova Kleplova T., Soukup T., Suchanek J. (2022). Human Platelet Lysate as an Efficient Supplement for In Vitro Expansion of Human Natal Dental Pulp Stem Cells. Biomolecules.

[29] Arpornmaeklong P., Sutthitrairong C., Jantaramanant P., Pripatnanont P. (2018). Allogenic human serum, a clinical grade serum supplement for promoting human periodontal ligament stem cell expansion. J. Tissue Eng. Regen. Med..

[30] Naskou M.C., Sumner S.M., Chocallo A., Kemelmakher H., Thoresen M., Copland I., Galipeau J., Peroni J.F. (2018). Platelet lysate as a novel serum-free media supplement for the culture of equine bone marrow-derived mesenchymal stem cells. Stem Cell Res. Ther..

[31] Kim E.Y., Lee J.B., Park H.Y., Jeong C.J., Riu K.Z., Park S.P. (2011). The Use of Embryonic Stem Cell Derived Bioactive Material as a New Protein Supplement for the In Vitro Culture of Bovine Embryos. J. Reprod. Dev..

[32] Allen M., Oberle K., Grace M., Russell A., Adewale A.J. (2008). A Randomized Clinical Trial of Elk Velvet Antler in Rheumatoid Arthritis. Biol. Res. Nurs..

[33] Yao B., Zhou Z., Zhang M., Leng X., Zhao D. (2021). Investigating the Molecular Control of Deer Antler Extract on Articular Cartilage. J. Orthop. Surg. Res..

[34] Chen J., Yang Y., Abbasi S., Hajinezhad D., Kontulainen S., Honaramooz A. (2015). The Effects of Elk Velvet Antler Dietary Supplementation on Physical Growth and Bone Development in Growing Rats. Evid.-Based Complement. Alternat. Med..

[35] Yu Y., Jin Y., Wang F., Yan J., Qi Y., Ye M. (2017). Protein Digestomic Analysis Reveals the Bioactivity of Deer Antler Velvet in Simulated Gastrointestinal Digestion. Food Res. Int..

[36] Ren C., Gong W., Li F., Xie M. (2019). Protective and Therapeutic Effects of Pilose Antler against Kidney Deficiency–Induced Osteoporosis. Cell. Mol. Biol..

[37] Pogozhykh O., Prokopyuk V., Figueiredo C., Pogozhykh D. (2018). Placenta and Placental Derivatives in Regenerative Therapies: Experimental Studies, History, and Prospects. Stem Cells Int..

[38] Mota-Rojas D., Orihuela A., Strappini A., Villanueva-García D., Napolitano F., Mora-Medina P., Barrios-García H.B., Herrera Y., Lavalle E., Martínez-Burnes J. (2020). Consumption of Maternal Placenta in Humans and Nonhuman Mammals: Beneficial and Adverse Effects. Animals.

[39] Rasool A., Alvarado-Flores F., O’Tierney-Ginn P. (2021). Placental Impact of Dietary Supplements: More Than Micronutrients. Clin. Ther..

[40] Cohen P.A., Bloszies C., Yee C., Gerona R. (2016). An amphetamine isomer whose efficacy and safety in humans has never been studied, β-methylphenylethylamine (BMPEA), is found in multiple dietary supplements. Drug Test. Anal..

[41] Buser G.L., Mató S., Zhang A.Y., Metcalf B.J., Beall B., Thomas A.R. (2017). Late-Onset Infant *Group B Streptococcus* Infection Associated with Maternal Consumption of Capsules Containing Dehydrated Placenta—Oregon, 2016. MMWR Morb. Mortal. Wkly. Rep..

[42] Saper R.B., Phillips R.S., Sehgal A., Khouri N., Davis R.B., Paquin J., Thuppil V., Kales S.N. (2008). Lead, Mercury, and Arsenic in US- and Indian-Manufactured Ayurvedic Medicines Sold via the Internet. JAMA.

[43] Tucker J., Fischer T., Upjohn L., Mazzera D., Kumar M. (2018). Unapproved Pharmaceutical Ingredients Included in Dietary Supplements Associated with U.S. Food and Drug Administration Warnings. JAMA Netw. Open.

[44] Brykman M.C., Goldman V.S., Sarma N., Oketch-Rabah H.A., Biswas D., Giancaspro G.I. (2022). What Should Clinicians Know About Dietary Supplement Quality?. AMA J. Ethics.

[45] White C.M. (2022). Continued Risk of Dietary Supplements Adulterated with Approved and Unapproved Drugs: Assessment of the U.S. Food and Drug Administration’s Tainted Supplements Database 2007–2021. J. Clin. Pharmacol..

[46] Jagim A.R., Harty P.S., Erickson J.L., Tinsley G.M., Garner D., Galpin A.J. (2023). Prevalence of Adulteration in Dietary Supplements and Recommendations for Safe Supplement Practices in Sport. Front. Sports Act. Living.

[47] Jairoun A.A., Shahwan M., Zyoud S.H. (2020). Heavy Metal Contamination of Dietary Supplement Products Available in the UAE Markets and the Associated Risk. Sci. Rep..

[48] Jonathan M.C., Li Z. (2018). Experimental Methods for Studying Cellular Heme Signaling. Cells.

