# Supplementary material for: Blood in Capsules: Multi-Technique Forensic Investigation of Suspicious Food Supplement
Source: Molecules. 2025 Nov 29;30(23):4600. doi: 10.3390/molecules30234600 (PMC12693171; doi:10.3390/molecules30234600)
Supplement: Supplementary file 1 [file molecules-30-04600-s001.zip › molecules-3941048-supplementary.pdf]

Supplementary Material

Blood in Capsules: Multi-Technique Forensic Investigation of Suspicious Food Supplement

Paweł Rudnicki-Velasquez [1,*], Magdalena Popławska [1], Karolina Pioruńska [1], Marta Łaszcz [1], Małgorzata Milczarek [2], Anna Pogorzelska [2], Michał Karyński [1] and Agata Błażewicz [1]

[1] Falsified Medicines and Medical Devices Department, National Medicines Institute, Chełmska 30/34, 00-725 Warsaw, Poland; m.poplawska@nil.gov.pl (M.P.); k.piorunska@nil.gov.pl (K.P.); m.laszcz@nil.gov.pl (M.Ł.); m.karynski@nil.gov.pl (M.K.); a.blazewicz@nil.gov.pl (A.B.)

[2] Biomedical Research Department, National Medicines Institute, Chełmska 30/34, 00-725 Warsaw, Poland; m.milczarek@nil.gov.pl (M.M.); a.pogorzelska@nil.gov.pl (A.P.)

* Correspondence: rudnicki.pb@gmail.com; Tel.: +48-228412121

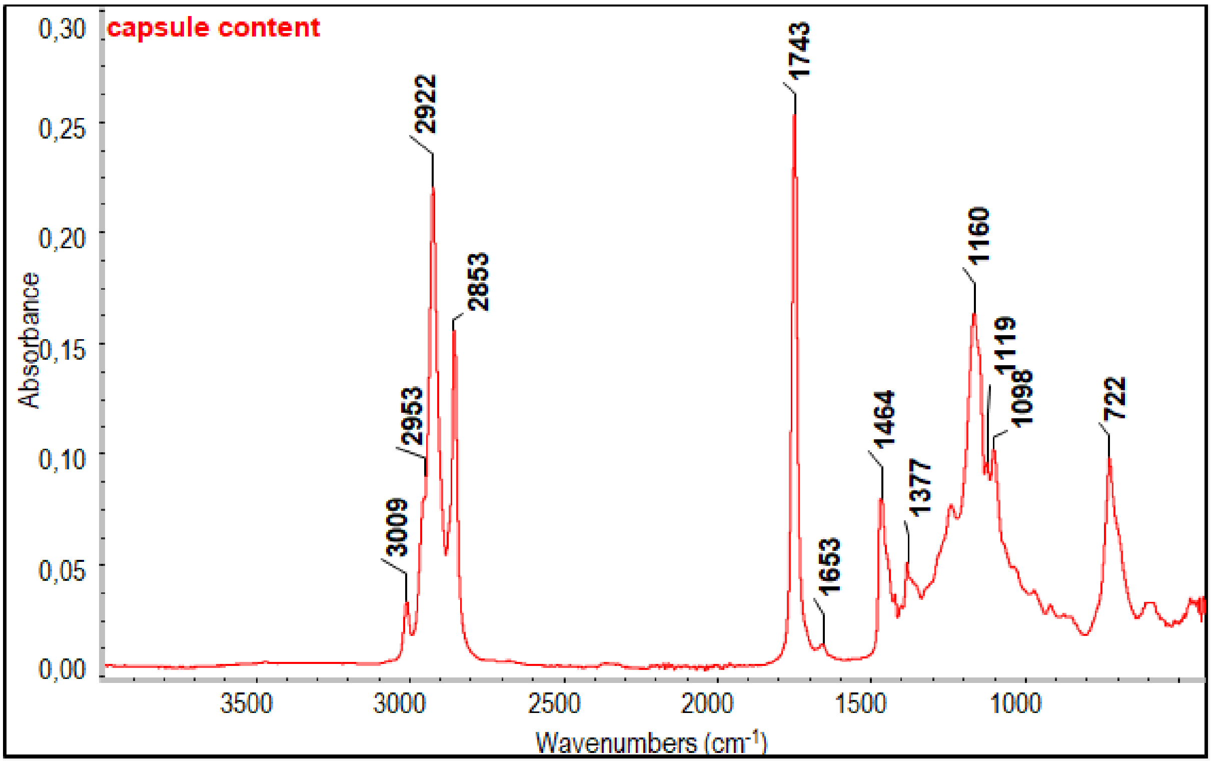

Figure S1. ATR-FTIR spectrum of the capsule content, showing characteristic absorption bands of triglycerides and other lipid constituents.

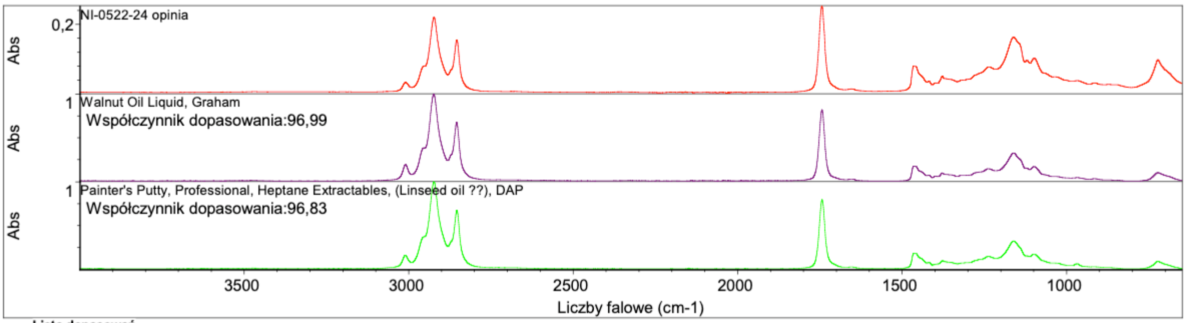

Lista dopasowań

	Nr indeksu	Wsp. dopasowania	Nazwa związku	Nazwa biblioteki
1	3684	96,99	Walnut Oil Liquid, Graham	HR Comprehensive Forensic FT-IR Collection
2	2328	96,83	Painter's Putty, Professional, Heptane Extractables, (Linsee	HR Comprehensive Forensic FT-IR Collection
3	1441	96,56	Grapeseed Oil, French	HR Comprehensive Forensic FT-IR Collection
4	3676	96,21	Vitamin A, 8000 I.U., USP, Fish Liver Oil in Soybean Oil and	HR Comprehensive Forensic FT-IR Collection
5	357	95,36	Almond Oil, Sweet, USA	HR Comprehensive Forensic FT-IR Collection
6	154	94,36	Glycerol trioleate	HR Specta Polymers and Plasticizers by ATR
7	1486	94,18	Hazelnut Oil, French	HR Comprehensive Forensic FT-IR Collection
8	2360	93,80	Peanut Oil, Roasted, Loriva	HR Comprehensive Forensic FT-IR Collection
9	2234	93,76	Olive Oil, (Spanish) Borges	HR Comprehensive Forensic FT-IR Collection
10	1621	93,50	EPOXIDIZED SOYBEAN OIL	HR Polymer Additives and Plasticizers

Figure S2. FTIR library match for the capsule liquid content, showing >96% similarity with walnut oil and other edible triglyceride matrices.

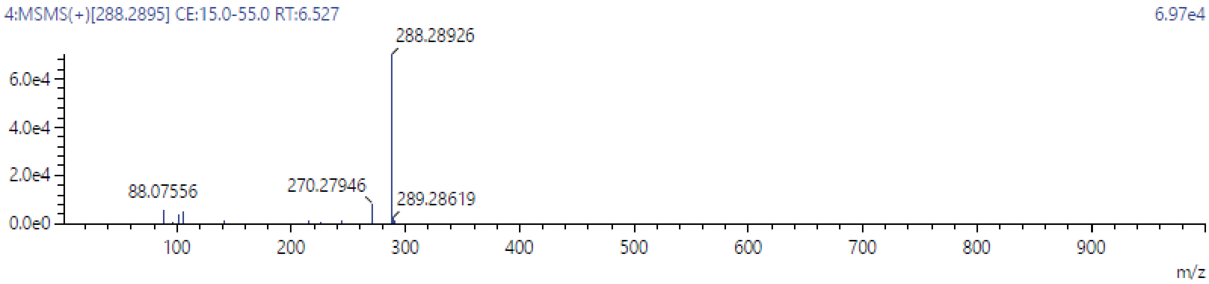

Figure S3A. Product ion spectrum of the peak at RT=6.527 min.

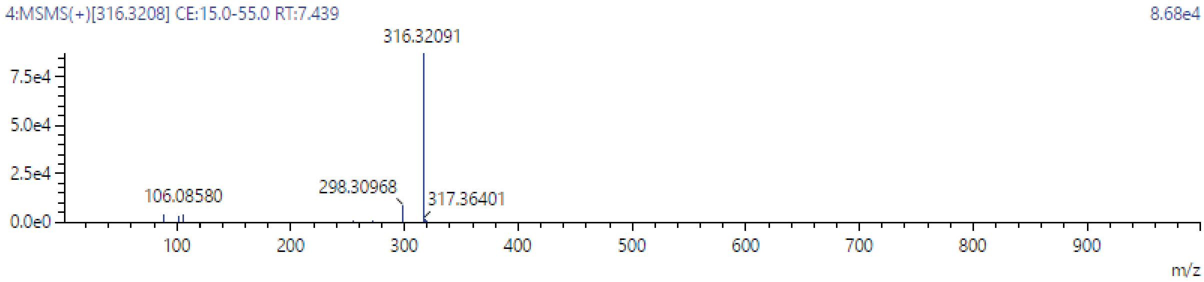

Figure S3B. Product ion spectrum of the peak at RT=7.439 min.

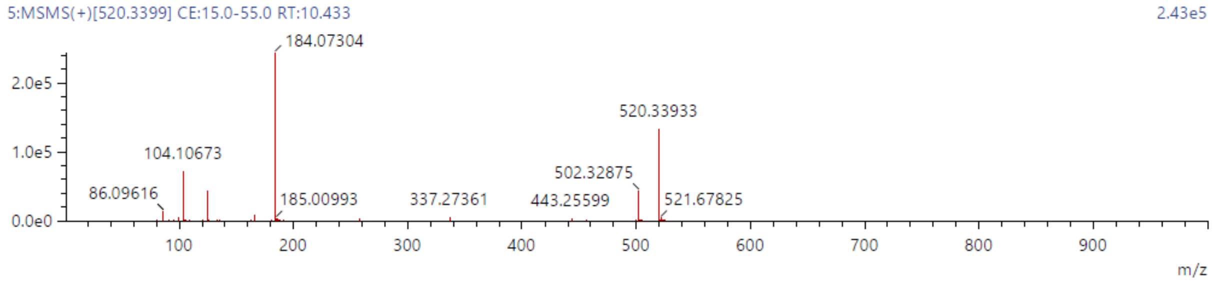

Figure S3C. Product ion spectrum of the peak at RT=10.433 min.

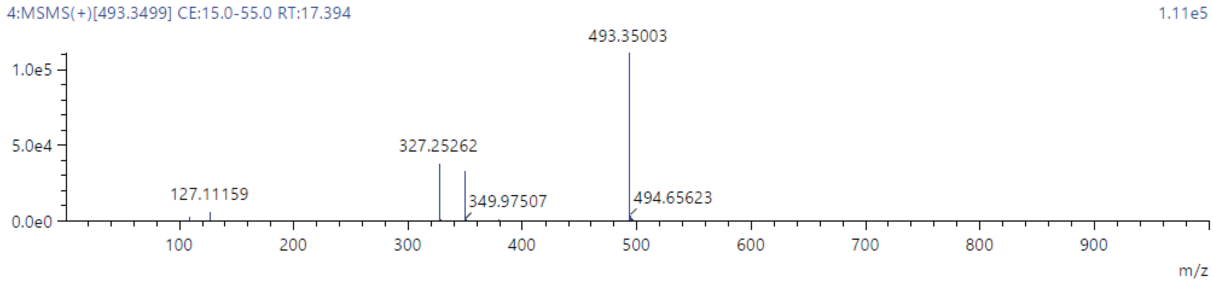

Figure S3D. Product ion spectrum of the peak at RT=17.394 min.

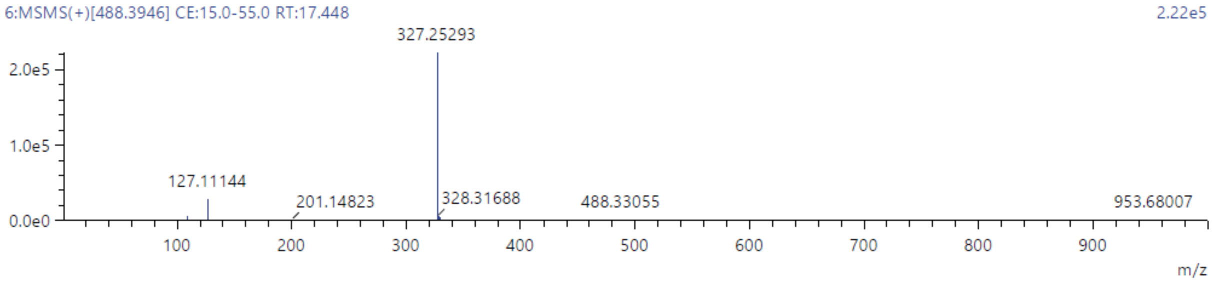

Figure S3E. Product ion spectrum of the peak at RT=17.448 min.

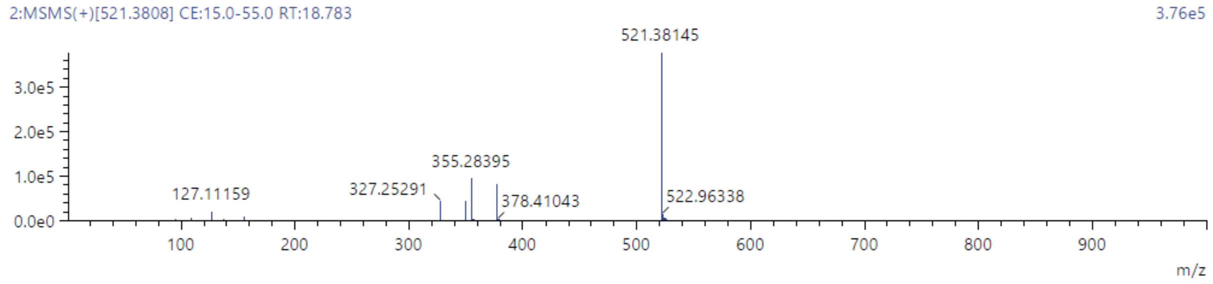

Figure S3F. Product ion spectrum of the peak at RT=18.783 min.

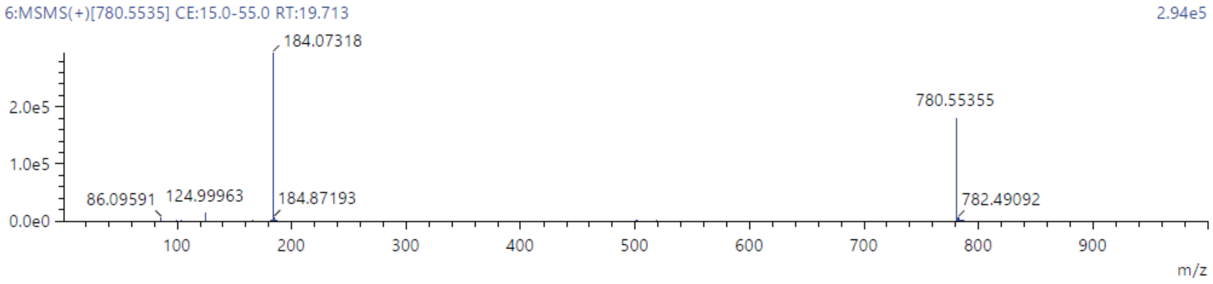

Figure S3G. Product ion spectrum of the peak at RT=19.713 min.

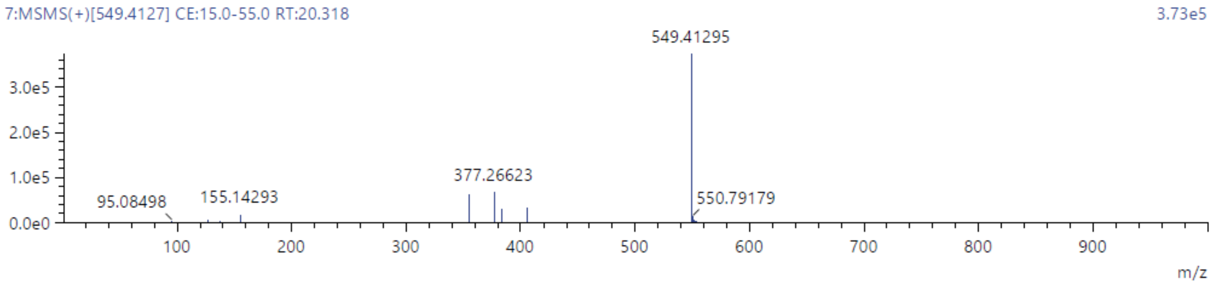

Figure S3H. Product ion spectrum of the peak at RT=20.318 min.

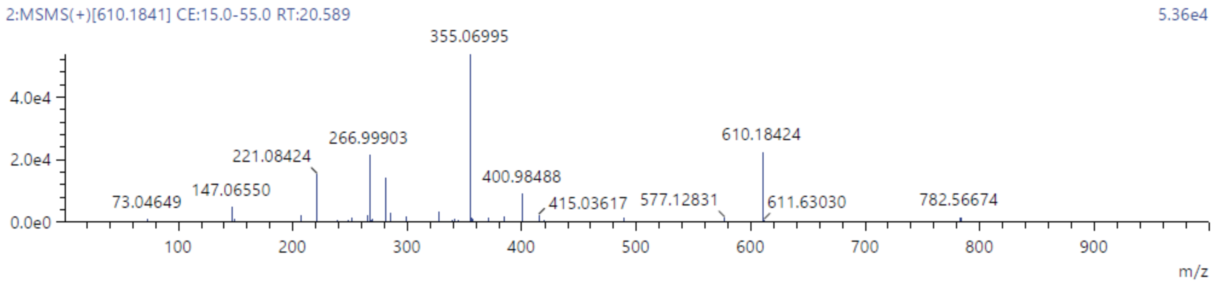

Figure S3I. Product ion spectrum of the peak at RT=20.589 min.

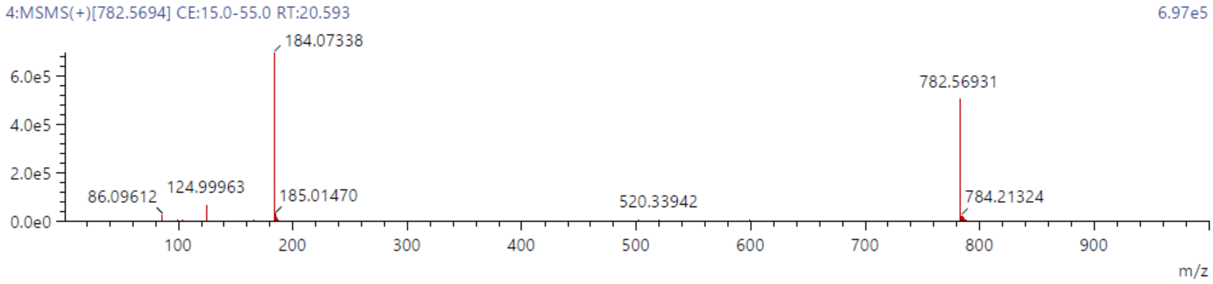

Figure S3J. Product ion spectrum of the peak at RT=20.593 min.

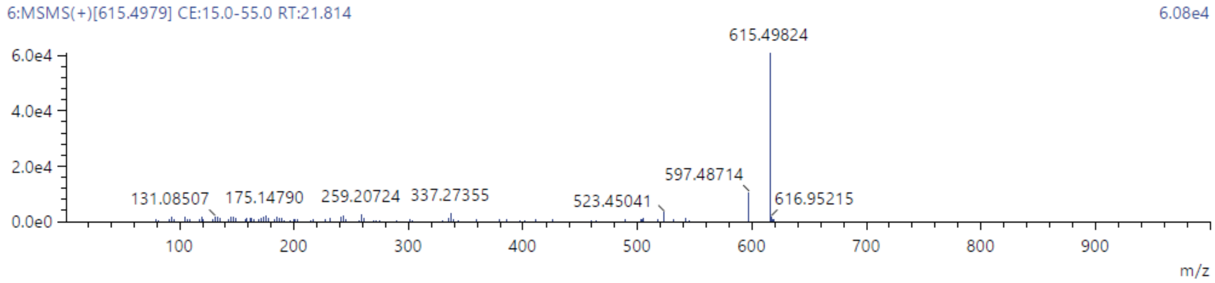

Figure S3K. Product ion spectrum of the peak at RT=21.814 min.

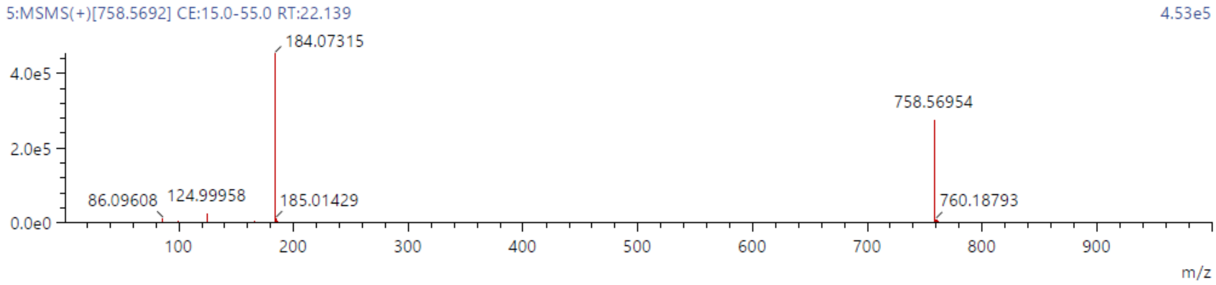

Figure S3L. Product ion spectrum of the peak at RT=22.139 min.

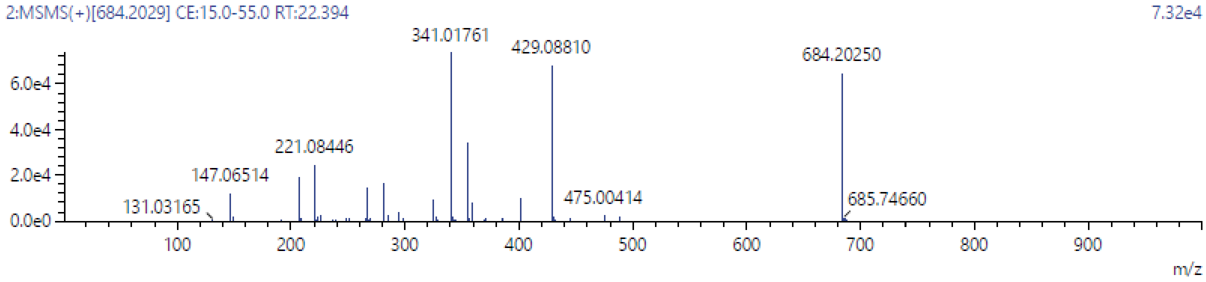

Figure S3M. Product ion spectrum of the peak at RT=22.394 min.

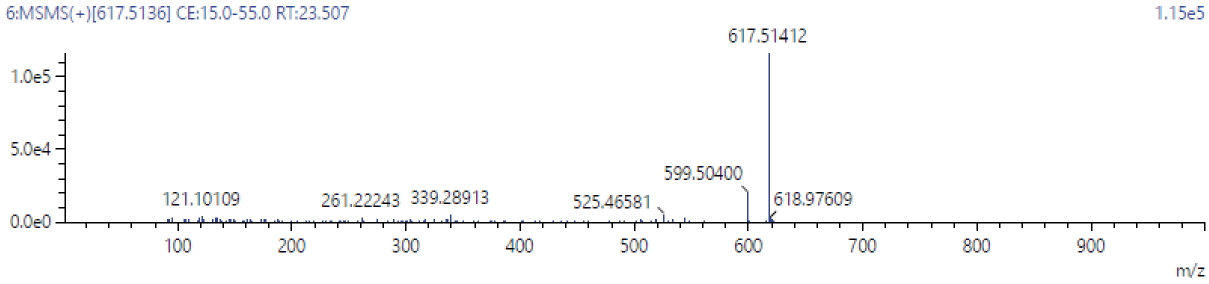

Figure S3N. Product ion spectrum of the peak at RT=23.507 min.

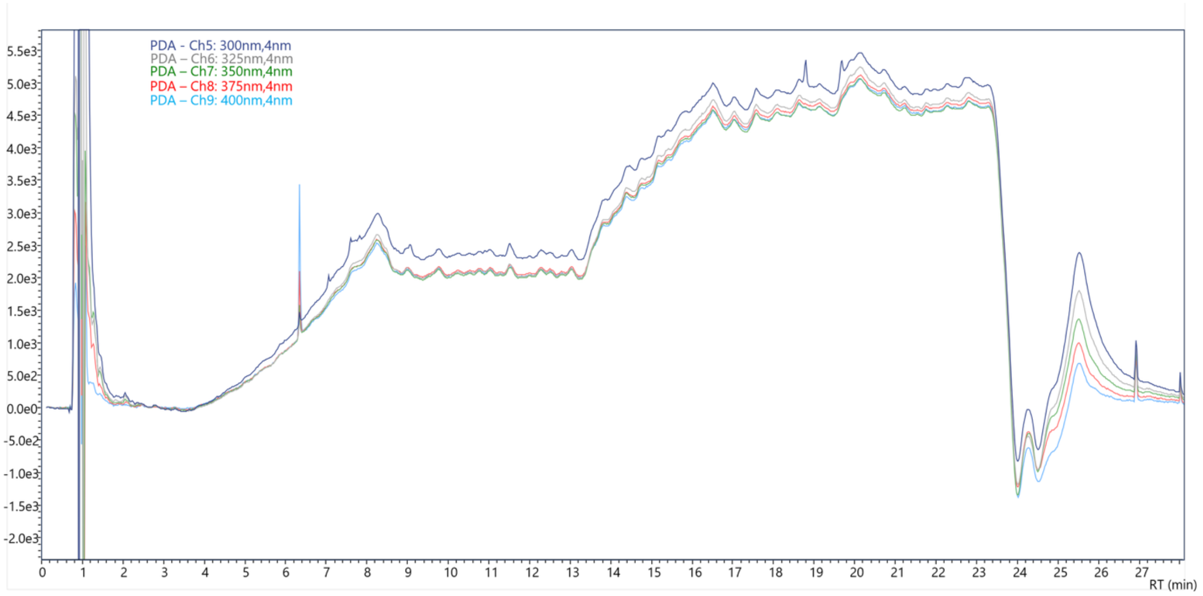

Figure S4. PDA chromatogram across the entire range of retention time with extracted wavelengths 300nm, 325nm, 350nm, 375nm, 400nm.
